# Meeting of the American Medical Association

**Published:** 1868-05-15

**Authors:** 


### *American Medical Association.*

This body convened at Washington a few days since and elected W. A. Balwin, of Alabama, President, and Caspar Wistar, of Philadelphia, Secretary. Our Reporter, expecting to be present, was not, and the details of proceedings have not yet come to hand. We are melancholy, but not without hope.

When medical societies are conducted as scientific associations, and not to bolster up hopelessly decayed apostles of Medical Reform, we shall take interest enough in them to be present either personally or by representative.

But speaking of Medical Reform, Why do not Prof. Gross and his *conferes* carry out into actual practice their "Teachers' Convention" programmes? Or are their resolutions only a tub thrown to the Apostolic whale? The JOURNAL pauses for a reply.

Since the above was written, the Boston *Medical Journal* has come to hand, from which we extract:

### MEETING OF THE AMERICAN MEDICAL ASSOCIATION.

Our national medical society has just held its annual meeting. Socially a success, it can hardly be considered so as a scientific re-union. Nor can we ever reasonably expect it to be otherwise. Its influence in removing sectional prejudices, in familiarizing the physicians of one part of our extended country with other parts, and with their professional brethren, is both great and salutary. It is, however, too widely extended, and holds too infrequent and too brief meetings to render its scientific proceedings choice or valuable. The great majority of our physicians are too busy in solving the practical problems of life and death daily presented to them, or too much occupied in the pursuit of individual aggrandizement and reputation, to become *savants*, or to devote themselves to the cultivation of pure science. It is a pity, too, that those unqualified to speak instructively, and those who delight in parliamentary quibbles, are allowed to take up the valuable time of a three days' annual session. Such, however, is the fate of many other large societies. The International Medical Congress in Paris was a signal instance of a like failure. The efforts of the National Association to raise the standard of medical requirements can not be too highly praised; and it is by this means only that the Society itself can ever become worthy of representing the whole nation.

---

The annual meeting was held at Washington, commencing on the morning of Tuesday, 6th inst., and adjourned on Friday, the 8th. After an address of welcome by Dr. Tyler, of Washington, the annual address was delivered by the President, Dr. S. D. Gross, of Philadelphia. The report of the Committee on Medical Education was ordered to be printed. The Committee on the President's Address reported several resolutions to carry out suggestions made in it, which were adopted. A resolution to establish nurse-training institutions in all large cities was referred to a special committee. The Committee on altering the Constitution advised many changes with regard to admission of members, etc. Some discussion was elicited by a resolution offered by the Committee on Medical Ethics, formally endorsing consultations with female practitioners who had received a regular medical education, and the subject was indefinitely postponed. Reports of various other committees were made and accepted.

W. A. Baldwin, of Alabama, was elected President; G. Mendenhall, Ohio; Noble Young, Washington; N. P. Munroe, Maine; and S. M. Bemis, Louisiana, Vice-Presidents; Casper Wistar, Philadelphia, Treasurer; and A. G. Semmes, Secretary for the ensuing year. New Orleans was appointed as the next place of meeting.

A number of committees were appointed, and it was "*Resolved*, that those gentlemen who wish to report on special subjects, and will pledge themselves to report at the next meeting, be requested to send their names and the subject they desire to report upon to the Secretary."

While in Washington, the delegates and members were received by the President, Chief Justice Chase, Speaker Colfax, and Senator Morgan, and on Wednesday evening the Army Medical Museum was thrown open for their inspection. After the adjournment on Friday, a large number of the members visited Mt. Vernon.

